# Supplementary material for: Sustained-input switches for transcription factors and microRNAs are central building blocks of eukaryotic gene circuits
Source: Genome Biol. 2013 Aug 23;14(8):R85. doi: 10.1186/gb-2013-14-8-r85 (PMC4054853; doi:10.1186/gb-2013-14-8-r85)
Supplement: Additional file 5 — HTML Browsable Motif Output. Zipped folder containing all WaRSwap and FANMOD motif output, viewable in a web browser. [file gb-2013-14-8-r85-S5.ZIP › HTML_browsable_motif_output/WaRSwap_ath_tair10/sigs_WSWRSLDraw_TAIR10-2500.pvals.heatmaps.html/motif_id_46_011101002_tftype_ath_upstream_-3000_0.html]

```
BG_MODEL = WARSwap
MOTIF_ID = 46_011101002
TF_TYPE = ath
UPSTREAM = -3000_0


PVals
FNR = 0.2	FNR = 0.4	FNR = 0.6	FNR = 0.8
deltaG = 60	0.031	0.018	0.003	0.783
deltaG = 70	0.031	0.022	0.002	0.766
deltaG = 80	0.027	0.021	0.006	0.768

ZScores
FNR = 0.2	FNR = 0.4	FNR = 0.6	FNR = 0.8
deltaG = 60	1.65	1.955	2.876	-0.906
deltaG = 70	1.625	1.932	2.84	-0.859
deltaG = 80	1.678	1.893	2.841	-0.855

StDevs
FNR = 0.2	FNR = 0.4	FNR = 0.6	FNR = 0.8
deltaG = 60	167820.661	78380.225	16596.342	1185.299
deltaG = 70	170547.131	79516.187	16852.683	1229.507
deltaG = 80	164419.92	80354.779	16886.06	1220.549
```
